# Genetic and antigenic characterization of recombinant nucleocapsid proteins derived from canine coronavirus and canine respiratory coronavirus in China

**DOI:** 10.1007/s11427-016-5038-1

**Published:** 2016-04-15

**Authors:** Shuai Lu, Yingzhu Chen, Kun Qin, Jianfang Zhou, Yongliang Lou, Wenjie Tan

**Affiliations:** 1 Institute of Medical Virology, Wenzhou Medical University, Wenzhou, 325035 China; 2 National Institute for Viral Disease Control and Prevention, Chinese Center for Disease Control and Prevention, Beijing, 102206 China

**Keywords:** canine coronavirus, canine respiratory coronavirus, nucleocapsid protein, expression, cross-reactivity

## Abstract

To characterize the antigenicity of nucleocapsid proteins (NP) derived from canine coronavirus (CCoV) and canine respiratory coronavirus (CRCoV) in China, the N genes of CCoV (CCoV-BJ70) and CRCoV (CRCoV-BJ202) were cloned from swabs obtained from diseased pet dogs in Beijing and then sequenced. The recombinant NPs (rNPs) were expressed in *Escherichia coli* and purified by nickel-affinity column and size exclusion chromatography. Sequencing data indicated that the N genes of CCoV-BJ70 and CRCoV-BJ202 belonging to two distinctly different groups were relatively conserved within each subgroup. Sodium dodecyl sulfate polyacrylamide gel electrophoresis (SDS-PAGE) results showed that rNPs of CCoV and CRCoV were expressed efficiently and isolated with a final purity of over 95%. Western blot analysis revealed the rNP from CRCoV could cross-react with mice antisera against human coronavirus (HCoV-229E, NL63, OC43, HKU1), while rNP of CCoV had cross-reactivity with only anti-sera against viruses belonging to the same group (HCoV-229E and NL63). In summary, CCoV and CRCoV rNPs were successfully expressed in *E. coli* and showed antigenic cross-reactivity with antisera raised against human coronaviruses. These findings indicate that further serologic studies on coronavirus infections at the animal-human interface are needed.

## INTRODUCTION

Coronaviruses are enveloped, positive-stranded RNA viruses that are highly prevalent in animal and human populations (Woo et al., 2009). To our knowledge, six types of coronaviruses have been recognized to be pathogenic to humans: HCoV-NL63, HCoV-229E, HCoV-OC43, SARS-CoV, HCoV-HKU1 and MERS-CoV. Because of the high mutation frequency and broad host ranges of this virus, novel coronaviruses have emerged and evolved to cross species during the past decade, generating novel strains, serotypes and genotypes. Canine coronavirus (CCoV) and canine respiratory coronavirus (CRCoV) are common viral pathogens responsible for mild to severe enteritis or respiratory symptoms in dog populations (Nicola and Canio, 2008). CCoV infection was first reported in a German military dog with diarrhea in 1971 (Binn et al., 1974). Since then, CCoV has been determined to be epidemic globally, and dogs of all breeds and ages appear to be susceptible to infection (Costa et al., 2014; Erles and Brownlie, 2009; Lorusso et al., 2009; Naylor et al., 2002; Yachi and Mochizuki, 2006). In China, CCoV was isolated from the heart and gastrointestinal contents of a sick dog for the first time in 1995 (Kong, 2007), and identified in deceased giant pandas in 2009 (Gao et al., 2009). In 2003, a novel respiratory coronavirus (CRCoV) distinct from CCoV was detected from the respiratory tracts of diseased dogs in the UK (Erles et al., 2003). Currently, CRCoV is found in dogs in Europe as well as Asia (Lorusso et al., 2009; Yachi and Mochizuki, 2006).

Coronaviruses are classified phylogenetically into four genera: Alphacoronavirus, Betacoronavirus, Gammacoronavirus, and Deltacoronavirus. Alphacoronaviruses include human coronaviruses HCoV-229E and HCoV-NL63, porcine transmissible gastroenteritis virus (TGEV), feline coronavirus (FCoV), and CCoV. Four human coronaviruses HCoV-OC43, SARS-CoV, HCoV-HKU1, MERS-CoV and bovine coronavirus (BCoV), as well as CRCoV, are Betacoronaviruses, but belong to different subgroups. Phylogenetically, CCoV is closely related to HCoV-229E and HCoV-NL63, while CRCoV is close to BCoV and HCoV-OC43. It has been suggested that CRCoV might share the same ancestor in this clade, or originated from a host transfer from bovine to canine (Erles et al., 2003).

Coronavirus harbors the largest genome among the known RNA viruses, about 27–31 kb, mainly encoding for the structural proteins spike (S), envelope (E), membrane (M), and nucleocapsid (N), and several other open reading frames (ORFs) encoding non-structural proteins. As reported, the glycosylated S protein mediates attachment of the coronavirus to cellular receptors and fusion with the cell membrane (Belouzard et al., 2012). The N protein is a phosphorylated protein, and is the most abundant. It interacts with viral RNA and makes up the core structure of viral particles (Li et al., 2005). It has been revealed that a high level of IgG antibodies against the N protein of SARS-CoV can persist for a long time after the onset of symptoms, and thus may be a marker for highly sensitive serological diagnosis (Tan et al., 2004). As the N protein is an unglycosylated protein, it can be easily expressed in bacteria and isolated with high purity. Therefore, antibodies against N protein appear to be ideal candidates as diagnostic markers.

Due to the high homology among coronaviruses in the same subgroup, antisera from HCoV-NL63 (Alphacoronavirus, subgroup 1b), HCoV-OC43 (Betacoronavirus, subgroup 2a), SARS-CoV (Betacoronavirus, subgroup 2b) and MERS-CoV (Betacoronavirus, subgroup 2c)-infected humans recognize the N proteins of a particular subgroup but do not react with N proteins from other subgroups, as previously reported (Sudhakar et al., 2014). However, cross-reactivities have been found previously among different genera. Anti-CCoV sera reacted with the N protein of SARS-CoV, a Betacoronavirus (Sun and Meng, 2004; Vlasova et al., 2007). Furthermore, polyclonal antibodies from Alphacoronaviruses, including HCoV-229E, feline infectious peritonitis virus (FIPV), and TGEV, reacted with SARS-CoV infected Vero cells (Shi et al., 2003), demonstrating conserved antigenic sites of N protein might exist among different genera. However, there is no data on the reactivity between CCoV or CRCoV N proteins and antisera derived from other human coronaviruses (such as HCoV-OC43, -NL63, -HKU1 and MERS-CoV).

Considering the global spread of CCoV and CRCoV infections among dog populations and the possible interspecies transmission from animals to humans, it is necessary to develop rapid and accurate assays for early diagnosis and epidemic control. However, there are very limited studies on CCoV and CRCoV circulating in China, and the antigenic relationship among CCoV, CRCoV and human coronaviruses. Here, the N coding genes of CCoV and CRCoV in China were amplified and sequenced, and then the recombinant N proteins of CCoV and CRCoV were prepared and characterized. In addition, cross-reactivity was analyzed between the recombinant proteins and polyclonal mice antisera from human coronaviruses.

## RESULTS

### Cloning and sequence analysis of NPs derived from CCoV and CRCoV in China

The genes encoding the N proteins of CCoV and CRCoV were amplified and sequenced with the gene lengths being 1,149 bp for CCoV-BJ70 and 1,347 bp for CRCoV-BJ202. Phylogenetic analysis showed that the CCoV N gene obtained in this study had the closest relationship with CCoV-NTU336, an Alphacoronavirus strain isolated in Taiwan (Figure 1). Amino acid sequence alignment revealed the N gene of CCoV-BJ70 had a relatively high identity of 98.2% compared to CCoV-NTU336. In addition, the N gene of

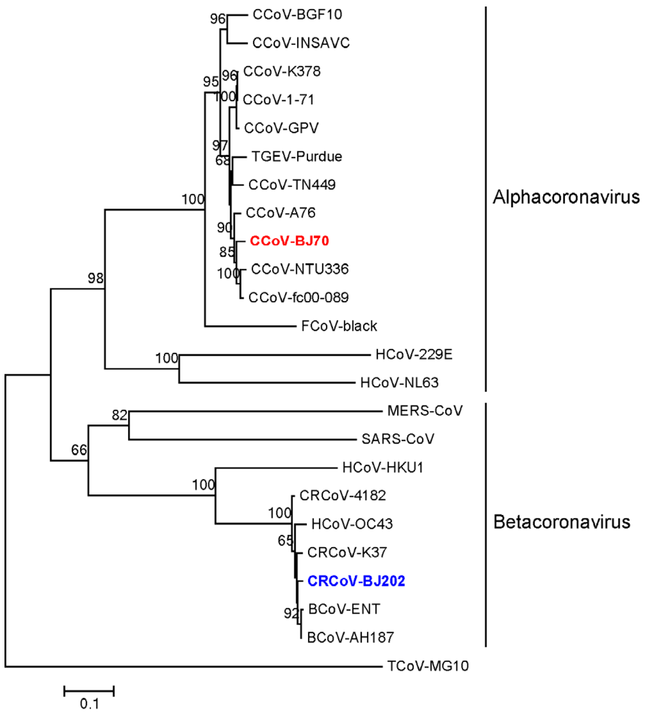

**Figure 1** Phylogenetic relationship of coronaviruses based N gene.

CCoV-BJ70 was also 91.4% to 97.7% identical to other strains of canine coronaviruses, including CCoV-TN449, CCoV-1-71, CCoV-fc00-089, CCoV-GPV, CCoV-A76, CCoV-K378, CCoV-BGF10, and CCoV-INSAVC. Compared with the closely related coronavirus strains from pig (TGEV-Purdue) and cat (FCoV-Black), the identity of amino acid sequence was 93.5% and 78.6% respectively (data not shown), showing the higher genetic relatedness between CCoV and TGEV. In order to determine the genotype of CCoV-BJ70, we amplified a partial sequence of the S gene and found that the genotype of CCoV-BJ70 belonged to CCoV-II (data unpublished). Comparison of the CRCoV-BJ202 N gene sequence to related CRCoV strains revealed that it was most similar to sequence data from BCoV-ENT, CRCoV-K37 and HCoV-OC43, which all belong to Betacoronavirus genus. The identity of the analyzed amino acid sequence was 98.4% to CRCoV-4182 and 97.6% to CRCoV-K37 respectively (data not shown), followed with the identity of 97.3% to BCoV-ENT and 96.7% to HCoV-OC43, while it was only 24.7% to CCoV-BJ70 (Table 1).

Furthermore, a highly conserved region (FYYLGTGP) was found through multiple alignments between the two N protein amino acid sequences to other five human coronaviruses (Figure 2). The N of CCoV-BJ70 was closest to that of HCoV-NL63 with 39% identical residuals, whereas only 24.5% to 27.0% with coronaviruses belonging to the Betacoronavirus genus. In contrast, the homology of N proteins between CRCoV-BJ202 and HCoV-OC43 can be up to 96.7%, but only 26.9% and 24.2% to HCoV-229E and HCoV-NL63, respectively (Table 1).

### Expression and purification of the recombinant N proteins of CCoV and CRCoV

Both recombinant NPs of CCoV and CRCoV were expressed in either soluble form or inclusion bodies, with obvious bands at about 45 and 55 kD respectively, which corresponds to our prediction (Figure 3). Optimization of the expression conditions indicated that N proteins had the highest soluble expression level with induction at 37°C for 4 h (data not shown). The soluble rNPs were purified by $Ni^{2+}$ and S75 columns. SDS-PAGE showed the isolated CCoV-N and CRCoV-N both had a high purity of over 95%. Meanwhile, Western blot analysis demonstrated reactivity with His monoclonal antibody specifically at the corresponding sizes (Figure 4).

### Antigenic relationships between N proteins of CCoV, CRCoV and HCoVs

Since the N proteins of coronaviruses are relatively conserved, we aimed to determine whether the N protein was responsible for antigenic cross-reactivity. Western blot analysis showed that the CCoV N protein cross-reacted weakly with mice antisera against the NP of HCoV-NL63 and 229E, indicating the N protein of CCoV may share the same antigenic epitopes with that of human alphacoronaviruses. However, the CRCoV N protein can react strongly with mice polyclonal sera against NP of HCoV-HKU1, OC43, NL63 and 229E, while having no reactivity with that of MERS-CoV (Figure 5).

## DISCUSSION

Since the emergence of the SARS-CoV in 2002, which was considered a zoonosis coronavirus transmitted from bats to humans (Peiris et al., 2003), more attention has been focused on the emerging and re-emerging viral pathogens in animals and humans. As viral pathogens commonly detected in diseased dogs worldwide, CCoV and CRCoV are thought to be responsible for gastrointestinal inflammations

**Table 1** Identity analysis of N protein among coronavirus strains in this research

| Coronavirus | CCoV-BJ70 | HCoV-229E | HCoV-NL63 | HCoV-OC43 | CRCoV-BJ202 | HCoV-HKU1 | MERS-CoV |
|---|---|---|---|---|---|---|---|
| CCoV-BJ70 | 100% | 38.1% | 39.9% | 24.5% | 24.7% | 25.9% | 27.0% |
| HCoV-229E | – | 100% | 48.8% | 27.1% | 26.9% | 29.2% | 25.7% |
| HCoV-NL63 | – | – | 100% | 24.8% | 24.2% | 25.6% | 25.5% |
| HCoV-OC43 | – | – | – | 100% | 96.7% | 65.8% | 33.0% |
| CRCoV-BJ202 | – | – | – | – | 100% | 66.1% | 33.0% |
| HCoV-HKU1 | – | – | – | – | – | 100% | 32.6% |
| MERS-CoV | – | – | – | – | – | – | 100% |

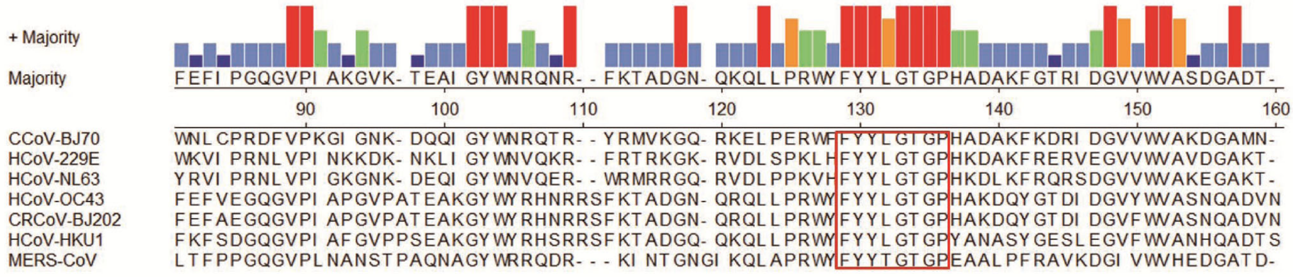

**Figure 2** Sequence alignment of N protein from seven different coronavirus strains. The highly conserved sequence is indicated by a red frame.

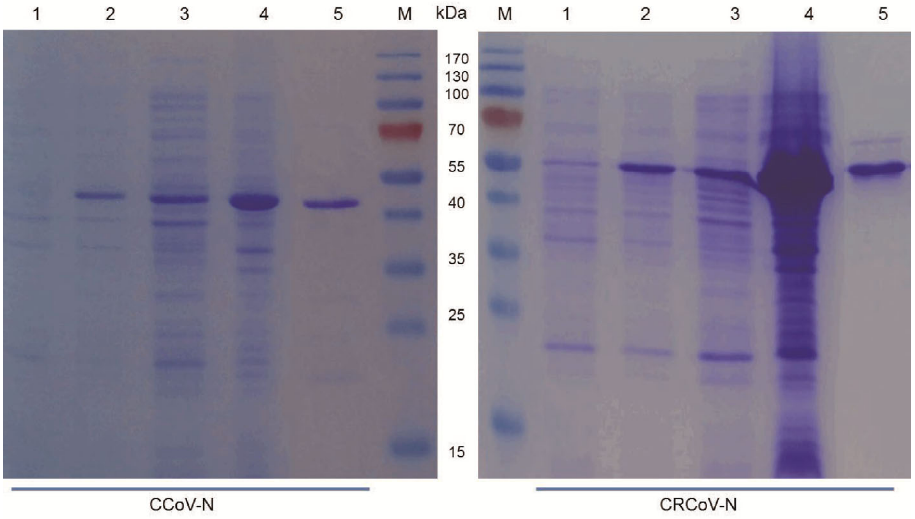

**Figure 3** SDS-PAGE analysis of the expression and purification of CCoV and CRCoV N proteins in *E. coli*. Lane M, marker. Lane 1, uninduced *E. coli*. Lane 2, induced by IPTG. Lane 3, supernatants of expression products. Lane 4, pellets of expression products. Lane 5, purified protein.

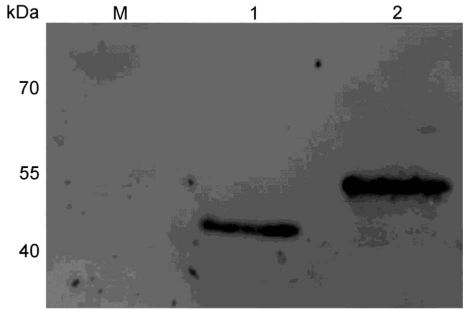

**Figure 4** Western blot analysis of recombinant proteins reacted with anti-His mouse monoclonal antibody. Lane M, marker. Lane 1, CCoV-NP. Lane 2, CRCoV-NP.

and respiratory infections, and signs of infections vary from mild to moderate, but are severe in puppies or in combination with other pathogens (Kong, 2007). N protein is an abundant and crucial structural protein in coronavirus particles, and has an important role in the viral immune response (Li et al., 2005). Antigen-capture enzyme-linked immunosorbent assays (ELISAs) using monoclonal antibodies against the N protein of SARS-CoV have been validated as useful for early diagnosis of SARS (Che et al., 2004). Thus, serological assays targeting the NP of coronaviruses are considered important to for diagnosis of viral infection. Characterization of the antigenicity of nucleocapsid proteins derived from individual coronaviruses is essential for the development of serological assays for coronaviruses.

It is notable that CoVs are characterized as having high rates of mutation and recombination, which is important to the transfer of hosts and alterations of virulence. We cloned the NP coding genes of CCoV (CCoV-BJ70) and CRCoV (CRCoV-BJ202) from diseased pet dogs in Beijing and followed with sequence analysis. Our data identified that CCoV-BJ70 and CRCoV-BJ202 belong to two distinctly different subgroups with close relationships with strains isolated from relatively near regions, based on the N gene phylogenetic tree. The CCoV-BJ70 clustered with other CCoVs in the Alphacoronavirus genus, including a Chinese strain isolated from deceased giant pandas, but was distinct from heterologous coronaviruses HCoV-229E and NL63. CRCoV-BJ202 was closely related to a Korean strain: CRCoV-K37, as well as HCoV-OC43. Furthermore, N protein identity between CCoVs and HCoVs also confirmed the phylogeny. Notably, the N protein identity observed between CCoV-BJ70 and HCoVs was only 25.5% to 39.9%, but was highly similar with other CCoV strains. However, CRCoV-BJ202 has a high identity of 96.7% to HCoV-OC43, suggesting that CRCoV might share the same origin with HCoV-OC43 or recombination has occurred between them. Although the phylogenic relationship and amino acid sequence similarity was observed, the potential of cross-reactivity between these coronaviruses is still unclear.

In this study, we expressed the N proteins of CCoV and CRCoV for serological studies. To our knowledge, the cloning and sequencing of the NP of CRCoV was first reported in China. Furthermore, we analyzed the antigenic relationship among CCoV, CRCoV and HCoVs. As shown in Figure 5, CCoV NP reacted with antisera from HCoV-229E and NL63 belonging to Alphacoronavirus, but showed weaker antigenicity, which may be due to the limited number of conserved antigenic sites among the subgroup. No significant cross-reactivity was observed to polyclonal antisera from three other HCoVs in the Betacoronavirus genus, indicating an extremely low avidity of the respective antibody, or a distinctly far antigenic relationship between these different clades. In contrast, CRCoV NP showed cross-reactive binding to antisera from mice immunized with the N proteins of HCoV-HKU1 and OC43,

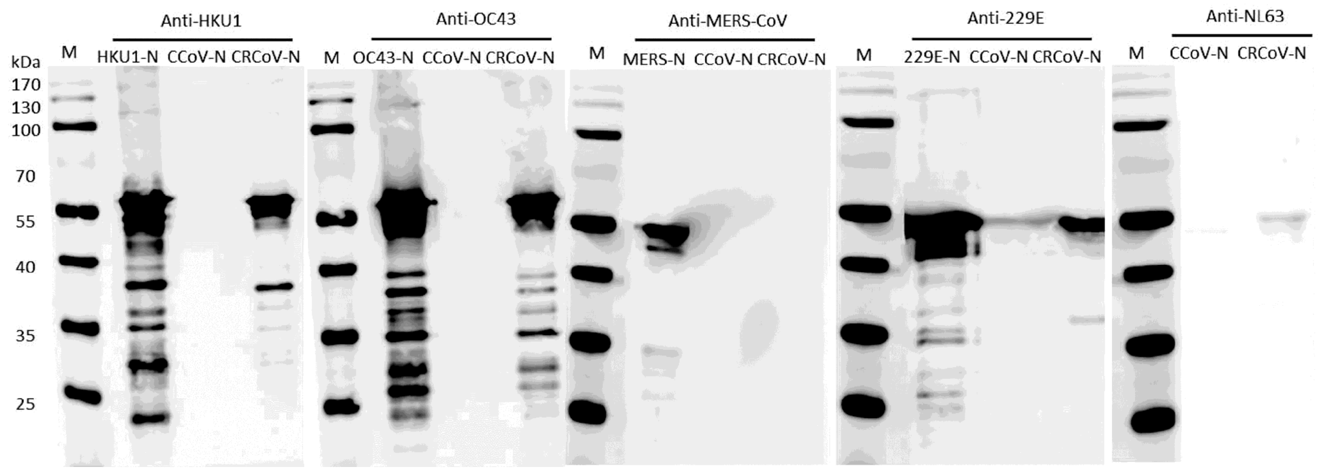

**Figure 5** Western blot analysis of cross-reactivity of CCoV and CRCoV N proteins with mice antisera against NP of human coronaviruses.

respectively, to a nearly same degree, and they all resolved into several fuzzy bands, except to the anti-MERS-CoV. Interestingly, CRCoV seems to be phylogenetically distant with HCoV-229E and NL63, but showed weaker reactivity than that of HCoV-HKU1 and OC43. This might be explained by conserved NP immune epitopes shared between both alpha and beta viruses, such as the highly conserved motif FYYLGTGP. In addition, CRCoV NP reacted strongly with anti-OC43 as did anti-HKU1, which may be attributed to the high identity of the NP amino acid sequence of CRCoV to HCoV-OC43 and HKU1 respectively. However, CRCoV NP showed weaker reactivity with anti-MERS-CoV than antisera derived from four other HCoVs. This may be due to the variations in their primary sequence, which can influence the antigenic sites and lead to a variety of cross-reactivity. Furthermore, the cross-reactivity generated between the two rNPs and anti-HCoVs was inconsistent with the identity of the NP sequences. This difference is possibly due to insufficient recognition by HCoVs antibodies, or different antigenic sites within the N proteins were exposed to an immune response, also indicating the potential complexity in serological diagnosis.

Our results revealed the presence of cross-reactive epitopes in the N proteins within a particular subgroup and also among different subgroups, and raised potential concerns in serological assays based on N proteins, providing implications for further research on coronaviruses testing. Therefore, it is important to identify a type-specific immune reactive epitope to the N protein or other appropriate diagnostic markers against coronaviruses with no antigenic cross-reactivity for identification of the coronavirus infections.

## MATERIALS AND METHODS

### Swabs and antisera

Swabs of positive CCoV and CRCoV (as determined by an antigen captured rapid diagnostic strip) were collected from diseased pet dogs visiting the veterinary hospital affiliated to the College of Veterinary Medicine, China Agricultural University. The anti-His monoclonal antibody was purchased from ZSGB-Bio (China). Mice antisera against the individual NPs of HCoV-229E, NL63, OC43, and HKU1 were kindly provided by Prof. Jianwei Wang and Li Guo (Chinese Academy of Medical Sciences). Mouse antisera against MERS-CoV were prepared in our lab.

### Cloning of the N genes from CCoV and CRCoV

Viral RNA was obtained from 140 μL of clarified swab extract with a QIAamp Viral RNA mini kit (QIAGEN, Germany) following the manufacturer's instructions. One-step RT-PCR (QIAGEN) was performed to amplify the full-length N genes of CCoV and CRCoV using specific primers with enzyme cleavage sites *Bam*H I and *Xho* I (Table 2). The PCR products were cloned into the pMD19-T vector and verified by sequencing. The nucleotide sequences obtained were deposited to GenBank under the accession numbers KT852997 & KT852998.

Sequences of other related coronaviruses strains in this study were downloaded from GenBank (CCoV-171: KC175339; CCoV-INSAVC: D13096;CCoV-TN449: EF056485; CCoV-K378: KC175340; CCoV-GPV: AY436636; CCoV-BGF10: AY342160; CCoV-fc00-089: AB781805; CCoV-A76: JN856008; CCoV-NTU336: GQ477367; TGEV-Purdue: DQ811789; FCoV-Black: EU186072; BCoV-ENT: NC_003045; CRCoV-K37: JX860640; CRCoV-4182: DQ682406; HCoV-229E:NC_002645; HCoV-NL63: NC_005831; HCoV-OC43: NC_005147; SARS-CoV: NC_004718; HCoV-HKU1: NC_006577; MERS-CoV: NC_019843; TCoV-MG10: NC_010800). Multiple sequence alignment was carried out using the ClustalW and MegAlign software. The phylogenetic tree was constructed by a neighbor-joining method using MEGA 6.0 software.

Table 2 Primers for plasmids construction (enzyme cleavage site is underlined)

| Target gene | Primer | Nucleotides sequences(5′→3′) | Restriction enzyme |
|---|---|---|---|
| CCoV-N | P1 | CGCGGATCCATGGCCAACCAGGGACAAC | *Bam*H I |
| CCoV-N | P2 | CCGCTCGAGTTAGTTCGTTACCTCATC | *Xho* I |
| CRCoV-N | P3 | CGCGGATCCATGTCTTTTACTCCTGG | *Bam*H I |
| CRCoV-N | P4 | CCGCTCGAGTTATATTTCTGAGGTGTC | *Xho* I |

### Expression and purification of the recombinant N proteins

Plasmids containing the N gene of CCoV and CRCoV were digested by the corresponding restriction enzymes and inserted into the bacterial expression vector pET28a (Novagen, Germany). All constructs were confirmed by enzyme digestion and DNA sequencing. The recombinant plasmids were transformed into expression *E. coli* competent cells BL21 (DE3). The *E. coli* culture was induced by adding isopropyl β-D-1-thiogalactopyranoside (IPTG) at a final concentration ~0.5 mmol $L^{-1}$ and incubating at three different conditions: 37°C for 4 h; 25°C for 10 h and 18°C for 16 h. After induction, the bacterial cells were harvested and suspended in binding buffer (Tris, 50 mmol $L^{-1}$; NaCl, 500 mmol $L^{-1}$; pH 8.0), and lysed by sonication for 20 min in an ice water bath, followed by centrifugation at 34,900×*g* for 30 min at 4°C. The supernatants and pellets were collected separately, and analyzed by 12% SDS-PAGE.

The recombinant NPs in the supernatant were purified using a nickel-affinity column using the AKTA system, and further processed by size exclusion chromatography (Superdex 75 column, GE, USA). Briefly, the protein was eluted using different gradients of elution buffer (Tris, 50 mmol $L^{-1}$; NaCl, 500 mmol $L^{-1}$; imidazole, 500 mmol $L^{-1}$; pH 8.0). The concentrated peak eluates were then subjected to an equilibrated S75 column and the purified soluble recombinant NPs were analyzed by SDS-PAGE.

### Antigenic analysis of the recombinant N proteins

The purified rNPs of CCoV or CRCoV separated by electrophoresis on the gels were transferred to nitrocellulose membrane, followed by blocking in 5% skim milk in phosphate buffer saline (PBS) at room temperature for 2 h. Afterwards, the blots were washed and incubated with anti-His monoclonal antibody (1∶1,000 dilution) and mice antisera against HCoV-229E, NL63, OC43, HKU1 and MERS-CoV (1∶200 dilution) separately at 4°C overnight. After three washes, goat anti-mouse IgG conjugated with HRP (1∶5,000 dilution, ZSGB-Bio) or IRDye anti-mouse IgG (1∶5,000 dilution, Odyssey) was added respectively and incubated at room temperature for 2 h. The membrane was thoroughly washed and then developed with the substrate DAB or using the Odyssey™ infrared imaging system.

**Compliance and ethics** *The author(s) declare that they have no conflict of interest.*

**Acknowledgements** *We thank Professor Jianwei Wang and Li Guo (Chinese Academy of Medical Sciences) for providing mice antisera against the individual NPs of HCoV-229E, NL63, OC43, and HKU1; We also thank the clinical staff in the veterinary hospital affiliated to the College of Veterinary Medicine, China Agricultural University for providing the swabs of positive CCoV and CRCoV. This work was supported by the Mega project for Infectious Disease Research of China (2014-ZX10004001-002, 2013ZX10004601), and National Basic Research Program of China (2011CB504704). The funders had no role in study design, data collection and analysis, decision to publish, or preparation of the manuscript.*

Belouzard, S., Millet, J.K., Licitra, B.N., and Whittaker, G.R. (2012). Mechanisms of coronavirus cell entry mediated by the viral spike protein. Viruses 4, 1011–1033.

Binn, L., Lazar, E., Keenan, K., Huxsoll, D., Marchwicki, R., and Strano, A. (1974). Recovery and characterization of a coronavirus from military dogs with diarrhea. Proccedings of the Annual Meeting of the USA Animal Health Assoc 78, 359–366.

Che, X., Hao, W., Wang, Y., Di, B., Yin, K., Xu, Y., Feng, C., Wan, Z., Cheng, V.C.C., and Yuen, K. (2004). Nucleocapsid protein as early diagnostic marker for SARS. Emerg Infect Dis 10, 11947–11949.

Costa, E.M., de Castro, T.X., Bottino Fde, O., and Garcia Rde, C. (2014). Molecular characterization of canine coronavirus strains circulating in Brazil. Vet Microbiol 168, 8–15.

Erles, K., and Brownlie, J. (2009). Sequence analysis of divergent canine coronavirus strains present in a UK dog population. Virus Res 141, 21–25.

Erles, K., Toomey, C., Brooks, H.W., and Brownlie, J. (2003). Detection of a group 2 coronavirus in dogs with canine infectious respiratory disease. Virology 310, 216–223.

Gao, F., Hu, G., Xia, X., Gao, Y., Bai, Y., and Zou, X. (2009). Isolation and identification of a canine coronavirus strain from giant pandas (Ailuropoda melanoleuca). J Vet Sci 10, 261.

Kong, Q. (2007). Progress on immune prevention of canine coronavirus disease (in Chinese). Prog Vet Med 28, 72–76.

Li, Y., Li, J., Liu, X., Wang, L., Li, T., Zhou, Y., and Zhuang, H. (2005). Detection of the nucleocapsid protein of severe acute respiratory syndrome coronavirus in serum: comparison with results of other viral markers. J Virol Methods 130, 45–50.

Lorusso, A., Desario, C., Mari, V., Campolo, M., Lorusso, E., Elia, G., Martella, V., Buonavoglia, C., and Decaro, N. (2009). Molecular characterization of a canine respiratory coronavirus strain detected in Italy. Virus Res 141, 96–100.

Naylor, M.J., Walia, C.S., McOrist, S., Lehrbach, P.R., Deane, E.M., and Harrison, G.A. (2002). Molecular characterization confirms the presence of a divergent strain of canine coronavirus (UWSMN-1) in Australia. J Clin Microbiol 40, 3518–3522.

Decaro, N., and Buonavoglia, C. (2008). An update on canine coronaviruses: viral evolution and pathobiology. Vet Microbiol 132 221–234.

Peiris, J.S.M., Lai, S.T., Poon, L.L.M., Guan, Y., Yam, L.Y.C., Lim, W., Nicholls, J., Yee, W.K.S., Yan, W.W., Cheung, M.T., Cheng, V.C.C., Chan, K.H., Tsang, D.N.C., Yung, R.W.H., Ng, T.K., Yuen, K.Y., and members of the SARS study group. (2003). Coronavirus as a possible cause of severe acute respiratory syndrome. Lancet 361, 1319–1325.

Shi, Y., Yi, Y., Li, P., Kuang, T., Li, L., Dong, M., Ma, Q., and Cao, C. (2003). Diagnosis of severe acute respiratory syndrome (SARS) by detection of SARS coronavirus nucleocapsid antibodies in an antigen-capturing enzyme-linked immunosorbent assay. J Clin Microbiol 41, 5781–5782.

Sudhakar, A., Robin, G., Boyd, L.Y., Eric, F.D, Vineet, D.M, Rachel, L.G, Trevor, D.S, Lisa, E.G, Mark, R.D, Maria, Z., and Baric, R.S. (2014). Evaluation of serologic and antigenic relationships between Middle Eastern respiratory syndrome coronavirus and other coronaviruses to develop vaccine platforms for the rapid response to emerging coronaviruses. J Infect Dis 209, 995–1006.

Sun, Z., and Meng, J. (2004). Antigenic cross-reactivity between the nucleocapsid protein of severe acute respiratory syndrome (SARS) coronavirus and polyclonal antisera of antigenic group I animal coronaviruses_ implication for SARS diagnosis. J Clin Microbiol 42, 2351–2352.

Tan, Y., Goh, P., Fielding, B., Shen, S., Chou, C., Fu, J., Leong, H., Leo, Y., Ooi, E., Ling, A., Lim, S.G., and Hong, W. (2004). Profiles of antibody responses against severe acute respiratory syndrome coronavirus recombinant proteins and their potential use as diagnostic markers. Clin Diagn Lab Immunol 11, 362–371.

Vlasova, A.N., Zhang, X., Hasoksuz, M., Nagesha, H.S., Haynes, L.M., Fang, Y., Lu, S., and Saif, L.J. (2007). Two-way antigenic cross-reactivity between severe acute respiratory syndrome coronavirus (SARS-CoV) and group 1 animal CoVs is mediated through an antigenic site in the N-terminal region of the SARS-CoV nucleoprotein. J Virol 81, 13365–13377.

Woo, P.C., Lau, S.K., Huang, Y., and Yuen, K.Y. (2009). Coronavirus diversity, phylogeny and interspecies jumping. Exp Biol Med 234, 1117–1127.

Yachi, A., and Mochizuki, M. (2006). Survey of dogs in Japan for group 2 canine coronavirus infection. J Clin Microbiol 44, 2615–2618.

**Open Access** This article is distributed under the terms of the Creative Commons Attribution License which permits any use, distribution, and reproduction in any medium, provided the original author(s) and source are credited.
